# Innovative Methodologies for the Early Detection of Breast Cancer: A Review Categorized by Target Biological Samples

**DOI:** 10.3390/bios15040257

**Published:** 2025-04-17

**Authors:** Antonella Grasso, Vittorio Altomare, Giulia Fiorini, Alessandro Zompanti, Giorgio Pennazza, Marco Santonico

**Affiliations:** 1 Breast Unit, Policlinico Campus Bio-Medico di Roma, 00128 Rome, Italy; a.grasso@policlinicocampus.it (A.G.); v.altomare@policlinicocampus.it (V.A.); 2 Unit of Electronics for Sensor Systems, Department of Science and Technology for Sustainable Development and One-Health, Università Campus Bio-Medico di Roma, 00128 Rome, Italy; giulia.fiorini@unina.it (G.F.); m.santonico@unicampus.it (M.S.); 3 Unit of Electronics for Sensor Systems, Department of Engineering, Università Campus Bio-Medico di Roma, 00128 Rome, Italy; a.zompanti@unicampus.it

**Keywords:** biosensor technologies, non-invasive cancer detection, early detection

## Abstract

Innovative biosensor technologies are revolutionizing cancer detection by offering non-invasive, sensitive, and rapid diagnostic tools, addressing the limitations of conventional screening. Non-invasive samples like breath, saliva, urine, and sweat, analyzed using advanced technologies like electronic nose systems and AI, show promise for early detection and frequent monitoring, though validation is needed. AI integration enhances data analysis and personalization. While blood-based methods remain the gold standard, combining them with less invasive sample types like saliva or sweat, and using sensitive techniques, is a promising direction. Conventional methods (mammography, MRI, etc.) offer proven efficacy, but are costly and invasive. Innovative methods using biosensors offer reduced infrastructure needs, lower costs, and patient-friendly sampling. However, challenges remain in validation, standardization, and low biomarker concentrations. Integrating both methodologies could create a comprehensive framework, combining reliability with accessibility. Future research should focus on robust biosensor development, standardization, expanding application to other cancers, exploring less-studied samples like sweat, and improving affordability for wider adoption, especially in resource-limited settings. The future lies in integrating diverse approaches for more sensitive, specific, and patient-friendly screening, improving early detection and outcomes.

## 1. Introduction

This introduction is divided into two sections: current epidemiological perspectives and state-of-the-art diagnostic methods.

### 1.1. Epidiemology and Current Challenges

**Figure 1:**
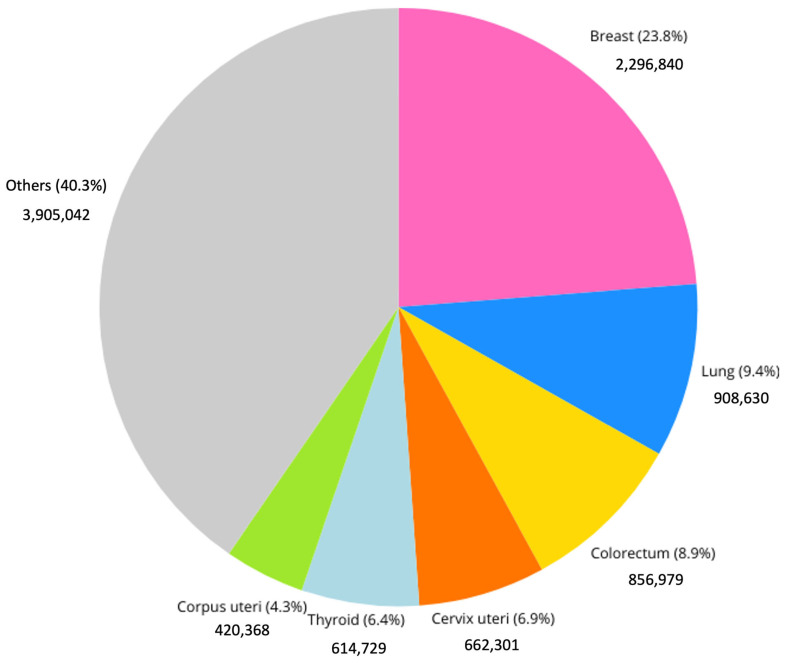
Incidence of different cancer types among females in 2022. “IARC, 2022” [1].

Breast cancer is the most frequently diagnosed cancer among women globally and a leading cause of cancer-related mortality. In 2022, approximately 2.3 million new cases (Figure 1) and 685,000 deaths were reported worldwide, accounting for 23.8% of all cancer cases and 15.4% of cancer deaths among women; moreover, an update from 2024 on the IARC site discusses the evidence suggesting that female breast cancer has surpassed lung cancer as the most diagnosed cancer worldwide. This is the reason why the early and timely diagnosis of breast cancer, along with comprehensive cancer treatment, is essential in many countries experiencing social and economic transition, where late-stage presentation remains widespread [1].

In Europe, around one in eight women will develop breast cancer during their lifetime, with a mortality rate of approximately 15% [2].

The incidence and mortality of breast cancer vary significantly across different regions, influenced by factors such as genetic predisposition, lifestyle, healthcare infrastructure, and public health policies.

In particular, as foreseen in the report of 2022 [2] and confirmed by the recent update in February 2025 [3], to effectively address disparities and track progress toward cancer control goals, countries with a low or medium Human Development Index (HDI) require improved cancer treatment and vital statistics, along with advancements in early detection and treatment access. Although 29 countries with a very high HDI have seen reductions in mortality rates, and 7 are already achieving the Global Breast Cancer Initiative target of an annual 2.5% decrease, the global burden remains uneven. By 2050, new breast cancer cases are projected to rise by 38% and related deaths by 68%, with the most significant impact present in low-HDI nations [3,4].

**Figure 2:**
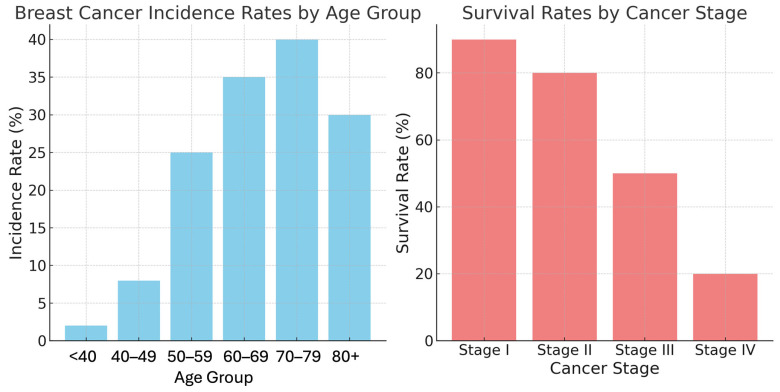
Breast cancer incidence rates by age group and survival rates by cancer stage [1].

The incidence and mortality rates of breast cancer are closely linked to age and stage at diagnosis (Figure 2). The risk of developing breast cancer increases with age, particularly after 50 years. The highest incidence rates are observed in the 70–79 age group. Although younger women (under 40 years of age) constitute a smaller proportion of breast cancer cases, they often present with more aggressive cancer subtypes [5]. The stage at which breast cancer is diagnosed significantly influences patient prognosis. Early-stage cancers (Stages I and II) generally have higher survival rates, while advanced stages (Stages III and IV) require more intensive treatments and have lower survival rates [6].

### 1.2. Conventional Methods for Breast Cancer Detection

Mammography, ultrasound, MRI, and PET scans are the current standard for breast cancer detection [7]. While crucial for early diagnosis, these methods suffer from significant drawbacks [8], including high costs, limited availability, and inconsistent accuracy, especially in younger women with dense breasts. Furthermore, the invasive nature of some procedures can discourage regular screening, hindering early detection and potentially impacting patient outcomes.

**Mammography**, the standard for breast cancer screening, is especially effective for women over 50. Using low-dose X-rays, it produces detailed breast tissue images and has proven to decrease breast cancer mortality. However, its effectiveness is reduced in women with dense breasts. Additionally, it involves low-level radiation exposure and carries a risk of false positives, leading to unnecessary biopsies [9].

**Magnetic Resonance Imaging (MRI)** uses magnetic fields and radio waves to create detailed images of breast tissue. It is especially useful for high-risk individuals, and can detect cancers in dense breasts [10], thus resolving some of the issues raised by mammography. However, MRI is more expensive, less accessible, and prone to more false positives, resulting in additional tests and biopsies [11], as already evidenced for mammography.

**Ultrasound** uses high-frequency sound waves to image breast tissue, and is a valuable complement to mammography, particularly for women under 50 and those with dense breasts [12]. While it avoids radiation and is generally well tolerated, thus overcoming MRI’s drawbacks, its accuracy is dependent on the operator’s skill, and it produces more false positives than mammography [13].

**Positron Emission Tomography (PET)** scans detect gamma rays from radioactive tracers that collect in areas of high metabolic activity [14]. Combined with CT scans, PET provides both functional and anatomical information, improving the accuracy of cancer staging. However, PET suffers from lower spatial resolution, complex image interpretation, and the limited availability of certain tracers [15].

Table 1 summarizes the pros and cons of the methods described above, highlighting the challenges that emerging technologies should address.

## 2. Emerging Diagnostic Methods

Given that early detection is strongly associated with improved survival rates and treatment outcomes in breast cancer, the development and implementation of effective diagnostic methodologies for early-stage identification are of paramount importance [16].

Early breast cancer detection significantly improves treatment success and long-term survival. These early-stage cancers are often less aggressive and more responsive to treatment, minimizing the need for extensive surgery and less toxic therapies, ultimately enhancing both survival rates and quality of life [17].

An extensive literature review was conducted to explore the various innovative methodologies developed for early breast cancer detection. This review categorizes the studies based on differences in sample types, techniques, technologies, and research objectives. The findings suggest a wide range of potential approaches and applications in the field. For each sample type, different technologies and methods can be used. In the following two sections, the sample types will be briefly presented and the different methodologies summarized, together with the different research objectives faced by the current literature. Then, the rest of the review will be organized into a series of paragraphs, each devoted to a specific sample type, reporting all the methods/technologies used for its analysis.

**Figure 3:**
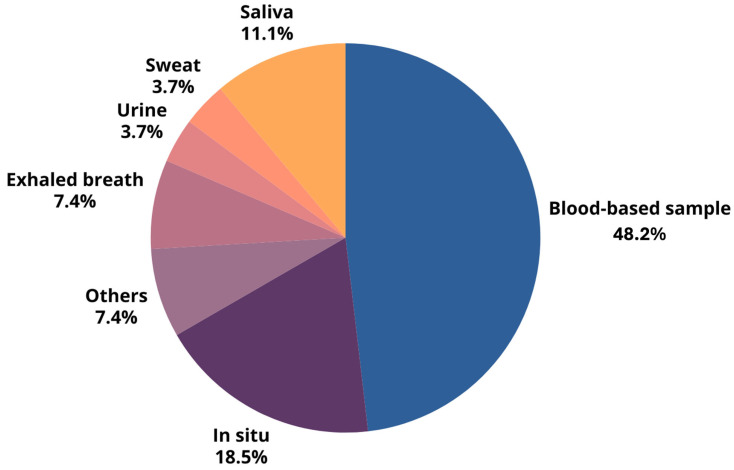
Percentage distribution of different sample types used in breast cancer detection, highlighting the predominance of blood-based samples and in situ methods.

Innovative breast cancer detection employs diverse sample types (see Figure 3 for the percentages of distribution). Blood-based samples are the most prevalent (48.1%) due to their accessibility and the wealth of systemic information they provide. These samples are frequently used in studies targeting specific biomarkers. In situ methods (18.5%), encompassing direct imaging and tissue sampling, offer detailed visualization of breast tissue alterations via techniques based on different sensors’ working principles, like piezoelectric analysis, ultrasound, tissue elasticity assessment, imaging, and thermal sensing. Exhaled breath analysis (7.4%) detects cancer-related metabolic shifts by analyzing volatile organic compounds (VOCs). Though less explored, non-invasive samples such as saliva (11.1%), urine (3.7%), and sweat (3.7%) hold promise and offer potential advantages for various diagnostic applications.

**Figure 4:**
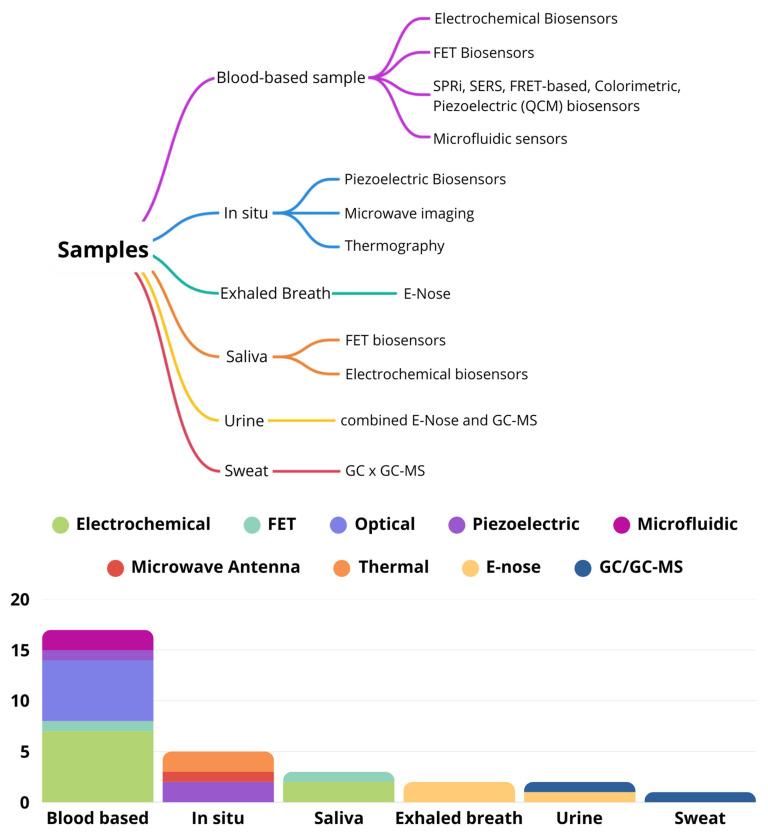
Different analytical techniques applied to different samples.

A variety of analytical techniques have been used across different sample types, with some methods suitable for multiple sample types. The chosen method depends on the specific research goals and the unique characteristics of each sample. This diverse approach is illustrated in Figure 4, which shows the range of analytical techniques applied to the various samples.

This literature review reveals several key research objectives driving the development of these innovative technologies. One major focus is identifying specific biological markers indicative of breast cancer, such as genetic biomarkers (genes and mRNA), protein biomarkers, cell lines, and cancer stem cell-associated biomarkers. Another objective is creating predictive models that analyze patterns and diagnose breast cancer based on diverse data inputs. These models can process large datasets, uncovering subtle patterns that improve early detection and diagnostic accuracy. Finally, some methods aim to detect and monitor structural and physiological changes in breast tissue. Techniques like piezoelectric sensors and microwave imaging offer non-invasive ways to identify abnormal tissue properties.

## 3. Analysis of Blood, Serum, and Plasma

Blood tests are a common tool in cancer research because they can detect a wide range of biomarkers. These biomarkers include genetic markers (like BRCA1 and miRNA-155), protein markers (such as HER2, MUC1, and CEA), and cell surface markers (like CD44 and ERα). Scientists use various techniques like electrochemical biosensors, optical and acoustic sensors, and microfluidic devices to study these biomarkers. These methods help to detect and measure the amount of biomarkers with different levels of accuracy.

### 3.1. Electrochemical Biosensors

Electrochemical biosensors stand out for their ability to accurately and sensitively detect biomarkers in blood. These devices utilize various signal transduction techniques, including impedimetric, voltammetric, and field-effect transistor (FET) methods, each offering unique advantages for biomarker detection.

Shahrokhian et al. created a highly sensitive **electrochemical DNA biosensor** for BRCA1 detection, as detailed in their study [18]. The biosensor utilizes electrochemically reduced graphene oxide and conducting polymers, and employs both voltammetric and impedimetric detection methods. This approach yielded a remarkable detection limit of 3 fM, crucial for identifying low-concentration genetic markers. While this design offers exceptional sensitivity, it also presents challenges, including complex electrode fabrication and the potential for non-specific binding. More recently, Mohammadpour-Haratbar et al. reported a series of electrochemical biosensors in which the use of graphene derivates (graphene oxide, reduced graphene oxide, and graphene quantum dots) increased the diagnostic performances in terms of LOD, lowering the concentration of detectable biomarkers and related compounds to challenging levels [19].

**Voltammetric biosensors** offer quantitative analysis by measuring the current changes resulting from redox reactions. For example, Hakimian et al. used cyclic voltammetry (CV) and a thiolated DNA probe-modified gold electrodes to detect miRNA-155. This method achieved a high sensitivity, with a detection range of 2.0 × 10^−20^ to 2.0 × 10^−12^ M. However, the stability of the DNA probes, which last about 1.5 months when stored at 4 °C, can be a limiting factor for long-term use [20]. In another study, Hu et al. developed a highly sensitive MUC1 **aptasensor using differential pulse voltammetry (DPV).** This aptasensor incorporated gold nanoparticles and horseradish peroxidase for signal amplification, achieving a low detection limit and a wide linear range. Testing in human serum samples demonstrated high sensitivity and specificity, with recoveries between 101.2% and 108.9%. Despite these promising results, aptamer synthesis and nanoparticle stability pose ongoing challenges [21]. Zhao et al. developed a novel **folding-based electrochemical aptasensor** for vascular endothelial growth factor (VEGF) detection using alternating current voltammetry (ACV). This sensor achieved a 5 pM (190 pg mL^−1^) detection limit in complex biological samples, even whole blood. Its high sensitivity, ease of preparation, and ability to operate in complex media make it a promising candidate for clinical use. However, aptamer degradation in serum could affect its long-term reliability [22]. For HER2 detection, Marques et al. used **linear sweep voltammetry (LSV) with a gold nanoparticle-modified electrode in a sandwich assay**. This method achieved a 4.4 ng/mL detection limit. While sensitive, it suffers from potential enzyme instability and a long assay time of approximately 2 h and 50 min [23]. Zhu et al. also focused on HER2 detection, creating a **square wave voltammetry (SWV) biosensor with a nanocomposite-modified gold nanoparticle electrode**. Their sensor achieved a significantly lower detection limit of 0.037 ± 0.002 pg/mL, though the complex surface modification process presents a challenge [24]. Ribeiro et al. developed a novel **electrochemical biosensor** for detecting CA 15-3, employing poly(Toluidine Blue) as a molecularly imprinted polymer (MIP) receptor. This sensor achieved a detection limit below 0.10 U mL^−1^ in diluted artificial serum and demonstrated a linear response across a broad concentration range (0.10 to 100 U mL^−1^). This work highlights the potential of combining MIPs with electrochemical detection for improved cancer biomarker detection, especially for point-of-care diagnostics [25]. Field-effect transistor (FET) biosensors hold great promise for cancer biomarker detection. Majd et al. designed an **FET biosensor for miRNA-155 detection** using MoS₂ as the conductive channel. Their design achieved an exceptionally low detection limit of 0.03 fM and exhibited high specificity, successfully differentiating between perfectly matched and single-base mismatched miRNA-155 sequences. While the biosensor showed good reproducibility, the stability of both the MoS₂ and the DNA probes requires further improvement for robust and reliable clinical application [26]. A recent comprehensive review [27,28] summarized the evidence and advancements in electrochemical biosensors that utilize advanced nanotechnologies for detecting breast cancer genes. These technologies include gold nanoparticle-reduced graphene oxide (AuNPs-GO), carbon nanotube-modified glassy carbon electrodes (CNT/GCE), zinc oxide nanowires (ZnONWs), carbon nanotube-modified screen-printed electrodes (SPEs), and reduced graphene oxide–yttrium nanocomposites (Y2O3–rGO/Apt/BSA). Specifically, regarding the detection of the HER2 protein, a more specialized review [29] highlighted recent advancements in the field. This review focused on the importance of detecting cancer early—whether at its onset, during recurrence, or while monitoring therapeutic interventions.

A detailed analysis of HER2 protein detection in serum was presented in another study, which examined two sensors utilizing gold sensor chips combined with amperometric detection of the enzyme label horseradish peroxidase (HRP). These biosensors/immunosensors were based on indirect sandwich enzyme-linked immunosorbent assays (ELISAs), where monoclonal antibodies (Abs) against HER-1 and HER-2 were attached to the sensors to capture the biomarkers [30]. Their performance was comparable to current standard techniques, although the overall process duration could and should be reduced.

To conclude the discussion on electrochemical biosensors, we find it useful to list their limitations and the challenges associated with them: complex fabrication; sensitivity to environmental factors; potential for non-specific binding; limited multiplexing capabilities; calibration and standardization issues; **dynamic range limitations;** regulatory hurdles; and stability issues.

### 3.2. Optical and Acoustic Biosensors

Optical and acoustic biosensors exploit changes in optical properties or acoustic waves upon target binding. These methods include Surface Plasmon Resonance Imaging (SPRi), Surface-Enhanced Raman Scattering (SERS), fluorescence, and colorimetric and piezoelectric sensors. Surface Plasmon Resonance (SPR) sensors are highly sensitive, label-free detectors that measure changes in the refractive index, making them ideal for identifying biomarkers in complex samples like blood. SPR’s adaptability allows for the tailoring of the sensor for specific biomarkers and detection needs by modifying the surface chemistry, functionalization, and detection environment. This versatility makes SPR effective for detecting a wide range of cancer biomarkers, as demonstrated by the studies presented below.

Szymańska et al. proposed that CEA could be a valuable biomarker for breast cancer detection, although their study was not specifically focused on this application. They developed an **SPRi sensor** utilizing cysteamine-modified gold chips for CEA detection, achieving a highly sensitive linear calibration curve with a detection limit of 0.1 ng/mL. However, the careful control of non-specific binding was required to ensure accuracy [31]. Erol et al. demonstrated the potential of molecularly imprinted polymers in SPR-based biosensors by developing a **nanoMIP–SPR sensor** for HER2 detection in human serum. Their approach exhibited a high affinity with and selectivity for HER2, while enabling rapid and cost-effective analysis without requiring signal amplification. This innovative design advanced SPR technology, achieving a detection limit of 11.6 pg/mL [32]. Verma et al. enhanced SPR technology by integrating a supervised machine learning approach (MLP regressor) to optimize sensor performance. They developed a **gold/TiO₂-coated photonic crystal fiber SPR sensor**, which exhibited exceptional sensitivity for MCF-7 breast cancer cells, achieving a maximum wavelength sensitivity of 11,034 nm/RIU. The addition of the gold/TiO₂ layer significantly improved the sensor’s detection capabilities, demonstrating the effectiveness of this novel approach [33]. Han et al. developed a **SERS-based sensor** for miR-K12-5-5p detection using Au/Ag hybrid porous GaN substrates, highlighting its potential for clinical diagnostics. Their approach demonstrated high sensitivity, with a detection limit of 884 pM, while the substrates exhibited promising uniformity and reproducibility. However, they emphasized the need for further improvements in stability to enhance long-term reliability [34]. Wang’s **FRET-based aptasensor** demonstrated high sensitivity for CEA detection, with limits of 7.9 pg/mL in aqueous solution and 10.7 pg/mL in human serum. However, the inherent complexities of the FRET system pose challenges for practical implementation [35]. Bai et al. developed a highly sensitive **colorimetric sensor** for BRCA1 detection, achieving an impressive detection limit of 10^−1^⁸ M. Their multiple-signal amplification strategy enhanced sensitivity, yet obtaining sufficient direct signals at low concentrations remains a challenge, indicating the need for further optimization [36]. Yang et al. developed a **quartz crystal microbalance (QCM) biosensor** targeting CD44 to assess the metastatic potential of breast cancer cells. The biosensor effectively distinguished between cells with different CD44 expression levels, achieving a detection limit of 300 cells mL^−1^ for MDA-MB-231 cells (high CD44 expression) and 1000 cells mL^−1^ for MCF-7 cells (lower CD44 expression). This differential sensitivity highlights its potential for evaluating metastatic potential. However, further optimization is necessary to improve its specificity and clinical applicability, as stability and reproducibility remain critical factors for its practical use [37]. The detection of CD44 expressing cancer cells was also recently performed using a fiber-optic ball resonator sensor via the measurement of the reflected light. The limit of detection obtained was 335 cells/mL [38]. The next challenge of this technique will be its application in vitro.

To conclude the discussion on optical and acoustic biosensors, we find it useful to list their limitations and the challenges associated with them: sensitivity to environmental factors; non-specific interactions; multiplexing limitations; stability concerns; sample interference; dynamic range limitations; and regulatory hurdles.

### 3.3. Microfluidic Devices

Microfluidic technologies offer a compact and efficient platform for biomarker detection by integrating multiple processes—sampling, dilution, reaction, separation, and detection—into a single chip.

Uliana et al. developed a fully **disposable microfluidic electrochemical device (μFED) for ERα detection in calf serum**, designed from low-cost materials for easy manufacturing. This innovative system achieved a remarkable detection limit of 10.0 fg mL^−1^, with high reproducibility, making it a promising candidate for point-of-care applications. In addition to improving reaction kinetics and minimizing sample and reagent consumption, the μFED offers significant advantages. However, the researchers noted that fabrication complexities could present challenges for large-scale adoption [39]. Gao et al. introduced a **high-throughput microfluidic platform for the simultaneous detection of multiple miRNA biomarkers** associated with breast cancer. Their system utilizes a self-assembled Poly-L-Lysine (PLL) substrate combined with microfluidic chips, enabling efficient multi-target analysis. By employing a three-segment hybridization approach, the platform allows for concurrent miRNA detection across various samples, achieving a detection limit of 1 pM with an impressive 30 min turnaround time [40]. Gao et al.’s microfluidic chip presents a more versatile and cost-effective alternative to single-target devices, like Uliana et al.’s ERα detector. By leveraging the charge polarity interactions of PLL for DNA probe immobilization, this design improves practicality for point-of-care applications. Its adaptability suggests a broader potential for on-site cancer diagnostics, making it a promising tool in the field.

To conclude the discussion on optical and acoustic biosensors, we find it useful to list their limitations and the challenges associated with them: fabrication complexity; sensitivity to environmental factors; and potential for the clogging of the channels.

### 3.4. Pros and Cons of Blood, Serum, and Plasma Samples

To conclude the illustration of the methods used for the analysis of blood, serum and plasma, general pros and cons are listed.

**Pros: High Biomarker Abundance**: Blood, serum, and plasma contain rich sources of biomarkers essential for diagnosing and monitoring disease progression [22,34]. **Early Detection**: These samples allow for the detection of biomarkers at early stages, crucial for timely intervention and improved survival rates [20]. **Variety of Detection Methods**: Techniques such as electrochemical biosensors, SPR sensors, and microfluidic devices can be employed, offering flexibility and sensitivity in biomarker detection [19,32]. **Stability of Samples**: Serum and plasma can be stored for extended periods, maintaining the stability of many biomarkers, which is advantageous for longitudinal studies [34].
**Cons: Invasiveness**: The collection of these samples can be uncomfortable for patients [33]. **Handling and Storage**: Proper handling and storage are required to prevent the degradation of the samples, which can affect the accuracy of the test results [34]. **Potential for False Positives**: There is a risk of cross-reactivity with non-target molecules, which can lead to false-positive results and unnecessary follow-up procedures [31]. **Complex Fabrication and Maintenance**: Advanced sensor technologies often require intricate preparation and maintenance, which can be resource-intensive and challenging to implement consistently across different settings [19,38]. **Variability in Biomarker Levels**: Biomarker levels can vary significantly between individuals and due to external factors, complicating result interpretation [38]. **Waste Management**: The disposal of biological waste must adhere to strict regulations, adding to operational complexity and costs [38]. **Cost and Resource-intensive Nature**: The collection and preparation of samples require resources and personnel, increasing operational costs, especially in mass screening contexts [34].

## 4. Analysis In Situ

In situ analyses are performed directly on tissues and include methods such as piezoelectric sensors, microwave imaging, and thermography.

### 4.1. Piezoelectric Sensors and Continuous Ultrasound Breast Monitor (cUSBr)

Piezoelectric sensors work by generating an electric charge in response to mechanical stress. When used for breast tissue analysis, they detect structural changes by translating pressure variations into electrical signals. This mechanism is extensively utilized in medical diagnostics and imaging to identify tissue abnormalities, facilitating the early detection and evaluation of potential health concerns.

The **cUSBr**, developed by Wenya Du et al. [41], enhances real-time breast tissue monitoring with its advanced imaging capabilities. This technology features a conformable ultrasound patch that employs a one-dimensional phased array based on piezoelectric principles, ensuring standardized and reproducible imaging. It offers a contrast sensitivity of approximately 3 dB, with axial and lateral resolutions of 0.25 mm and 1.0 mm at a depth of 30 mm, enabling the detection of cysts as small as 0.3 cm. Additionally, the cUSBr achieves a maximum imaging depth of 80 mm and provides a broader field of view than conventional handheld probes. Xu et al. developed a **high-performance piezoelectric sensor array** designed for breast cancer detection, achieving an accuracy rate of 88% [42]. Their research focused on assessing how factors such as DC voltage duration, application depth, and breast density influence detection sensitivity. By applying a direct-current (DC) voltage, the sensor detects tissue deformation, enabling the evaluation of tissue stiffness—an important indicator of potential tumors.

To conclude the discussion on piezoelectric sensors and cUSBr, we find it useful to list their limitations and the challenges associated with them: operator dependency, potential skin contact issues, long-term stability of materials, and time interval issues dependent on scanning operations.

### 4.2. Microvawe Imaging

By detecting differences in dielectric properties, microwave imaging can distinguish between normal and cancerous tissues. This cutting-edge technology offers a promising alternative for detecting breast cancer.

Elsheakh et al. investigated the use of machine learning techniques to enhance the performance of a microwave imaging system for breast cancer detection [43]. Support vector machines (SVM) and logistic regression classifiers were trained on datasets derived from microwave signals of 3D tumor models, enabling the effective classification of malignant tumors. Their study also introduced a **smart bra embedded with microwave textile-based antenna sensors**, achieving an impressive detection sensitivity of 85%. By integrating wearable technology with advanced imaging, this approach has the potential to facilitate more frequent and comfortable monitoring.

To conclude the discussion on microwave imaging, we find it useful to list its limitations and the challenges associated with it: this technology relies on advanced equipment, which may limit its availability in less-equipped clinical settings; the accuracy of detection is influenced by tumor size, with smaller tumors being more challenging to detect; additionally, patient compliance is crucial, otherwise, the effectiveness of monitoring can be compromised.

### 4.3. Thermography

Thermography detects cancerous tissues by measuring the higher temperatures they exhibit compared to normal tissues due to increased metabolic activity.

Elouerghi et al. designed a **flexible wearable thermography system** for early breast cancer detection, utilizing a network of bioheat microsensors [44]. The system features 28 miniaturized sensors arranged on a flexible star-shaped surface to cover the most sensitive breast areas. To validate the concept, the researchers conducted computer simulations and experimental tests on a breast phantom. The system successfully detected simulated tumors at different depths, recording temperature variations of up to 0.6 °C for tumors at 15 mm, 0.5 °C at 20 mm, and 0.45 °C at 30 mm. While the study does not report specific sensitivity and specificity values for this system, the authors reference previous studies indicating that breast thermography can achieve sensitivity and specificity rates of 95%. Future clinical evaluations are planned to assess the system’s actual diagnostic performance. Sree et al. investigated the **Cyrcadia Breast Monitor** (CBM), a thermal sensing device designed for breast cancer detection [45]. By continuously monitoring circadian temperature variations in breast tissue, the CBM provides significant advantages in identifying malignant tissues. Its non-invasive nature and ability to collect data over time make it a valuable tool for early detection. The recorded thermal data are analyzed using machine learning models, improving the accuracy of distinguishing between benign and malignant lesions. The study highlights the growing role of thermography in breast cancer detection, emphasizing the CBM’s potential in enhancing diagnostic capabilities.

To conclude the discussion on thermography, we find it useful to list its limitations and the challenges associated with it: the need for the robust calibration for reliable results; influence of environmental confounding factors; individual variability; limited data on tissue structure; and clinical acceptance and patient compliance.

### 4.4. Pros and Cons of In Situ Sample Analyses

To conclude the illustration of the methods used for the in situ analysis, general pros and cons are listed.

**Pros:**
**Real-time Monitoring**: Technologies such as thermography systems allow for continuous monitoring, which is useful for the early detection of breast cancer [46]. **Potential for Clinical Use**: These systems can be integrated into clinical settings, enhancing diagnostic capabilities and the timeliness of interventions [42]. **Detection of Anomalies**: Technologies like ultrasound can provide significant imaging, facilitating the identification of tissue abnormalities [36]. **Non-invasive Approach**: The proposed technologies are designed to be non-invasive, reducing patient discomfort and improving the acceptance of procedures [44].
**Cons:**
**Stringent Measurement Conditions**: Patients are required to comply with specific conditions, which may restrict the practical applicability of these technologies [42]. **Mechanical Challenges**: Geometric variability and the deformability of breast tissue can complicate the accuracy of analyses and measurements [44]. **Technical Limitations**: these two technologies have technical gaps, such as dependence on technician experience and issues with skin contact [44]. **Sensor Limitations**: The sensors used may have limited bandwidths, affecting the effectiveness of analyses and the capability for self-screening [45].

## 5. Exhaled Breath

Analyzing exhaled breath is becoming a promising non-invasive approach for detecting volatile organic compounds (VOCs) linked to breast cancer. By collecting and examining breath samples, this method can identify patterns that may indicate the presence of cancer.

### 5.1. Electronic Nose Technology

Breath analysis using electronic nose (E-Nose) technology has shown great potential in cancer detection. Yang et al. conducted a study utilizing an **E-Nose with 32 carbon nanotube sensors** to analyze the exhaled breath of 899 individuals, including 351 breast cancer patients [46]. Predictive classification models were developed using machine learning algorithms, specifically Random Forest, which achieved an impressive 91% detection accuracy, with 86% sensitivity and 97% specificity. These results highlight the promise of E-Nose technology for early breast cancer detection. However, certain limitations must be considered, such as the potential influence of anesthetics on VOC composition in intraoperative settings and the exclusion of smokers, which may affect sample diversity. Further research is needed to validate breath-based tests for breast cancer diagnosis in more diverse populations. Díaz de León-Martínez et al. (2020) conducted a study using an **E-Nose** for exhaled breath analysis, achieving an impressive 98% classification accuracy [47]. Despite these promising results, the study faced limitations, including a small sample size and potential environmental factors that could influence breath composition. Ref. [46] in the discussion and ref. [47] in the introduction propose two different lists of possible VOC biomarkers. This apparent contradiction accounts for extreme difficulties in identifying specific biomarkers, which opens the way for a VOC profile alteration study as an alternative strategy proposed by 46 and 47, which is based on exhaled breath fingerprinting via E-Nose-like instruments.

To conclude the discussion on E-Nose, we find it useful to list its limitations and the challenges associated with it: the environmental sensitivity of sensors, variability in breath composition due to personal factors, and the need for validation in larger, diverse populations

### 5.2. Pros and Cons of VOC Analysis in Breath Samples

**Pros:**
**Non-Invasive**: Breath analysis is a comfortable, unobtrusive method [48]; this characteristic makes it particularly suitable for frequent screening and monitoring. **Rapid Results**: E-Nose technology allows for quick analysis, providing timely diagnostic information [46].
**Cons:**
**Environmental Sensitivity**: Accuracy can be affected by environmental factors like temperature and humidity [46]. **Standardization Challenges**: Variability in breath composition due to personal factors complicates standardization [46]. Factors such as diet, medication, and smoking habits can influence VOC profiles, requiring robust normalization methods. **Need for Validation**: Further validation in larger, diverse populations is necessary to confirm the results [46,47].

## 6. Saliva

Saliva offers a non-invasive and convenient method for cancer detection, as its biomarkers can be correlated with those found in blood. Its easy collection process makes saliva an ideal option for frequent monitoring.

### 6.1. Field-Effect Transistor (FET) Biosensors

**Field-effect transistor (FET) biosensors** have emerged as a promising technology for detecting cancer biomarkers in saliva. Wan et al. [48] developed a highly sensitive saliva-based biosensor capable of detecting HER2 and CA15-3, with an impressive detection limit of 1 fg/mL. This innovative device integrates FET technology with disposable test strips, similar to those used for glucose monitoring, which were functionalized to selectively target these breast cancer biomarkers. By employing a synchronized double-pulse method, the biosensor achieved remarkable sensitivity levels of approximately 70/dec for HER2 and 30/dec for CA15-3. It also demonstrated a rapid testing time of under 15 ms and required only 3 μL of saliva, highlighting its potential for early breast cancer detection. Despite its operational simplicity and high sensitivity, challenges remain, such as ensuring specificity, standardizing sample collection, and assessing long-term stability. Further clinical validation with diverse patient cohorts is necessary to confirm its diagnostic accuracy and enhance its applicability in clinical settings. Wei et al. designed **integrated electrochemical sensors** capable of detecting both IL-8 mRNA and protein simultaneously, highlighting their potential for oral cancer diagnosis [49]. Similarly, Torrente-Rodríguez et al. developed an **amperometric platform** for IL-8 detection, demonstrating the feasibility of using saliva-based diagnostics for other cancers, including lung and pancreatic tumors [50].

A novel technique has demonstrated remarkable efficiency in detecting breast cancer biomarkers HER2 and CA15-3, utilizing commercially available disposable strips similar to common glucose detection strips [50]. Notably, this method achieves an exceptionally low detection limit of just 1 fg/mL, significantly surpassing the sensitivity of conventional enzyme-linked immunosorbent assays. Its advantages are further highlighted by a rapid testing time of under 15 ms and a minimal sample requirement of only 3 μL of saliva.

### 6.2. Pros and Cons of Saliva Analysis

**Pros**:
**Non-Invasive:** Saliva collection is simple, non-invasive, and painless, making it suitable for frequent monitoring and reducing patient discomfort [48]. **Cost-Effective:** The use of saliva-based tests can reduce the costs associated with more invasive procedures, making them more accessible for patients [49]. **Potential for Multiple Biomarkers:** Saliva can contain a variety of biomarkers, allowing for the simultaneous detection of multiple conditions [49,50]. **Lower Interference:** Saliva generally has lower levels of interfering substances compared to blood, which can simplify the analysis and improve the accuracy of the results.
**Cons**:
**Lower Biomarker Concentration:** Biomarker concentrations in saliva may be lower than those in blood, potentially requiring more sensitive detection methods to achieve reliable results [51]. **Variability:** Factors such as hydration, food intake, and oral health can influence saliva composition, leading to variability in the results and potentially affecting diagnostic accuracy [49]. **Standardization:** Standardized protocols for saliva collection and processing are essential to ensure reliable and reproducible results, which can be challenging to implement across different settings [50]. **Limited Research for Some Biomarkers:** While there is growing interest in saliva-based diagnostics, some biomarkers may not yet have established correlations with disease states, necessitating further research [48,49,50].

## 7. Urine

Urine contains various cancer-related metabolites and biomarkers, making it a simple and non-invasive diagnostic tool. However, its easy collection is offset by the challenge of detecting biomarkers present in low concentrations.

**Electronic nose (E-Nose)** technology has also demonstrated significant potential in urine analysis for cancer detection. Benet et al. developed an E-Nose system modeled on a dog’s olfactory capabilities, designed to detect volatile organic compounds (VOCs) in human urine. The study analyzed 90 urine samples from both breast cancer patients and control subjects, with the E-Nose prototype achieving a 75% classification rate, including 100% sensitivity, but only 50% specificity. To enhance accuracy, gas chromatography-mass spectrometry (GC-MS) data were used to train a convolutional neural network (CNN) algorithm, which improved the classification rate, taking it to 92.31%. The E-Nose utilized a sensor array to identify VOC patterns in urine, effectively distinguishing between cancerous and non-cancerous samples in a clinical setting [51].

### Pros and Cons of Urine Analysis

**Pros:**
**Non-Invasive**: Urine collection is simple, non-invasive, and painless, making it suitable for frequent monitoring. **Ease of Collection**: Urine samples can be collected easily without the need for specialized medical personnel or equipment. **Cost-Effective**: The use of urine-based tests can reduce the costs related to more invasive procedures.
**Cons:**
**Lower Biomarker Concentration**: Biomarker concentrations in urine may be lower than those in blood, potentially requiring more sensitive detection methods. **Variability**: Factors such as diet, hydration, and lifestyle can influence urine composition and affect the consistency of results. **Standardization Challenges**: Standardized protocols for urine collection and processing are essential to ensure reliable and reproducible results.

## 8. Sweat

Sweat analysis offers a non-invasive method for real-time biomarker monitoring, enabling continuous tracking without the need for invasive procedures. Although it is less commonly used for cancer detection than breath or saliva analysis, recent studies have started investigating the presence of volatile organic compounds (VOCs) in sweat. This emerging approach has the potential to expand non-invasive cancer screening options.

The study conducted by Leemans et al. focused on identifying breast cancer-specific VOCs in the sweat of patients [52]. To achieve this, the researchers collected sweat samples from the breast and hand areas of 21 breast cancer patients before and after tumor ablation. These samples were then analyzed using thermal desorption coupled with two-dimensional gas chromatography and mass spectrometry (GC × GC − MS), which allowed for the analysis of a total of 761 VOCs.

The study successfully detected at least 77 VOCs, revealing significant differences between the pre- and post-surgery states of the patients. By utilizing machine learning models, particularly logistic regression, the researchers identified specific VOCs that could distinguish between these states, achieving high sensitivity rates. However, the study faced limitations, including a small sample size and variability in VOC profiles due to individual differences and external factors.

### Pros and Cons of Sweat Analysis

**Pros:**
**Non-Invasive Collection**: Sweat sampling is a non-invasive method, making it more patient-friendly compared to blood or tissue samples. **Cost-Effective**: If validated, sweat analysis could serve as a low-cost alternative to traditional screening methods, particularly beneficial in resource-limited settings.
**Cons:**
**Limited Sample Size**: The small number of participants in the study may limit the applicability of the findings to the broader population. **Variability in VOC Composition**: Factors such as diet, hydration, and individual metabolism can influence sweat composition, leading to variability in VOC profiles. **Standardization Challenges**: There is a need for standardized protocols for sweat collection and analysis to ensure consistent and reliable results across different studies and populations.

## 9. Conclusions

Biosensor technologies are transforming cancer detection by providing rapid, sensitive, and non-invasive diagnostic tools. While these innovations enhance early detection and patient outcomes, they must also overcome the challenges of traditional screening methods. The use of diverse sample types—including blood, saliva, breath, urine, and sweat—offers flexibility in application, though each comes with its own advantages and limitations. As a summary to aid the discussion and conclusion, Table 2 presents a comparative overview of all the cited experiments, highlighting the key factors involved.

The shift toward non-invasive cancer detection is evident, with breath, saliva, urine, and sweat analyses emerging as promising options for patient-friendly and frequent monitoring. Although blood-based methods remain the gold standard due to their high biomarker abundance, research is increasingly focused on integrating multiple approaches. Applying highly sensitive detection techniques from blood analysis to less invasive samples like saliva or sweat could enhance diagnostic accuracy while minimizing limitations.

Advanced technologies, such as electronic nose systems and machine learning algorithms, are further supporting early detection, though standardization and validation remain necessary. Additionally, the integration of artificial intelligence across various detection methods is improving data analysis and interpretation, paving the way for more precise and personalized diagnostics. This trend is expected to continue and strengthen the overall effectiveness of breast cancer screening.

Traditional imaging techniques such as mammography, MRI, ultrasound, and PET remain the gold standard for breast cancer detection, providing direct tissue visualization and clinically validated accuracy. Their widespread adoption is supported by established protocols and integration within healthcare systems. However, these methods require substantial infrastructure, specialized facilities, and trained personnel, leading to high costs and limited accessibility. Additionally, patients often face challenges such as procedure invasiveness, radiation exposure, and long wait times for appointments and results.

In contrast, innovative approaches leverage non-invasive sampling—such as breath, saliva, sweat, and urine—alongside advanced biosensor technologies. These methods offer key benefits, including lower infrastructure requirements, reduced operational costs, rapid result delivery, and the potential for point-of-care or home-based testing. Their non-invasive nature enhances patient comfort and allows for more frequent monitoring.

Despite these advantages, novel methodologies still face hurdles in clinical validation, protocol standardization, and result consistency. Technical challenges, such as low biomarker concentrations and environmental influences, must also be addressed. Nevertheless, these innovations hold significant promise, particularly for early screening and continuous monitoring [54].

Recent advancements in biosensors and sensing technologies that utilize nanostructured materials are significantly transforming the landscape of traditional diagnostic tools. These innovations are driving the development of more practical, accurate, and efficient diagnostic platforms, making them increasingly suitable for real-world clinical applications. Nanostructured materials, in particular, have emerged as a cornerstone in this evolution due to their exceptional attributes—such as cost-effectiveness, superior sensitivity, versatility in detecting multiple targets (multimodal detection), and the ability to be easily miniaturized into compact devices.

Their unique physical and chemical properties make them especially promising for the detection of a wide range of analytes, including critical clinical targets like cancer biomarkers [55]. However, for these advanced diagnostic systems to deliver reliable and reproducible results, it is crucial that the nanomaterials employed exhibit a high degree of selectivity and binding efficiency. This means they must be engineered to specifically interact with target molecules while minimizing non-specific interactions that could interfere with the accuracy of the test. Achieving this level of specificity is essential to fully harness the potential of nanostructured materials in next-generation diagnostic solutions [56]. In the context of breast cancer diagnosis using nano-enabled biosensors, it is critically important to ensure that the nanomaterials employed are capable of binding to their target molecules with high specificity and efficiency. This selective binding is essential to accurately detect cancer-related biomarkers amidst the complex biochemical environment of biological samples. Minimizing non-specific interactions with unrelated molecules not only enhances the precision of the diagnostic process, but also significantly reduces the likelihood of false positives or negatives. Therefore, achieving a high degree of molecular recognition and binding fidelity is fundamental to the development of reliable, sensitive, and clinically viable diagnostic tools for early breast cancer detection [57].

With reference to SPR, which has been discussed in this review [31,32,33], a derivation of this method is the application of the Localized Surface Plasmon Resonance (LSPR) sensors, extensively explored for breast cancer diagnostics, and several studies demonstrate the effectiveness of LSPR-based technologies in detecting breast cancer biomarkers [53,58,59,60]. By enlarging the target to cancer biomarkers in general, excellent lower detection limits can be obtained, together with affordability and durability, by using 2D nano-engineered sensing materials [61,62].

A hybrid diagnostic model that integrates both conventional and innovative methods could create a more effective and accessible screening framework. By combining the proven reliability of traditional imaging with the convenience and affordability of emerging technologies, early detection rates could improve, making screening more widely available across diverse populations

Future research should prioritize overcoming the current limitations of biosensor technologies, particularly by improving their reproducibility and ensuring high specificity and sensitivity across diverse populations. Standardizing testing conditions—especially for breath- and sweat-based biosensors—is essential for reducing environmental and physiological variability and enhancing result reliability.

Expanding biosensor applications beyond breast cancer detection presents another promising avenue. Developing multiplexed biosensors capable of detecting multiple biomarkers simultaneously could provide a more comprehensive diagnostic profile and improve early cancer detection across various types. Additionally, applying detection techniques that have proven to be effective in traditional samples, such as blood, to less studied sample types like sweat, could enable non-invasive and continuous monitoring, combining high sensitivity with patient-friendly sampling methods.

Enhancing the affordability and accessibility of these technologies is also critical, particularly for resource-limited regions. The development of point-of-care devices that integrate multiple detection methods could significantly expand the reach of breast cancer screening programs, making early diagnosis more widely available.

While significant progress has been made, continued research and innovation remain crucial to refining these diagnostic tools. The future of breast cancer detection lies in integrating diverse approaches to create more sensitive, specific, and patient-friendly screening methods, ultimately improving early detection rates and patient outcomes.

Roadmap

By concluding with a roadmap for a future outlook in the field, some points can be assessed:

- Traditional imaging—such as mammography, MRI, ultrasound, and PET—remains essential for breast cancer detection, offering validated accuracy, but requiring costly infrastructure and trained personnel [63]. These methods also come with drawbacks such as invasiveness, radiation, and long wait times;
- In contrast, non-invasive approaches using biosensors and bodily fluids offer lower costs, faster results, and better accessibility, including potential home testing. Despite challenges like low biomarker levels and inconsistent results, these innovations show strong promise for early detection and continuous monitoring [64];
- The shift toward non-invasive cancer detection is gaining momentum, with breath, saliva, urine, and sweat offering patient-friendly options for frequent monitoring. While blood tests remain the gold standard due to high biomarker levels, research is increasingly exploring multi-sample integration. Applying sensitive blood detection methods to non-invasive samples could boost accuracy, with fewer drawbacks;
- Technologies like electronic noses and machine learning support early detection, though standardization and validation are still needed. AI integration is improving analysis and interpretation, moving diagnostics toward greater precision and personalization;
- The integration of unobtrusive sensor technology for monitoring and frequent examination could favor early detection and early intervention in a collaborative synergy between robotics and sensors [65].

## Figures and Tables

**Table 1:** Pros and cons of conventional methods for breast cancer detection.

Method	Pros	Cons
**Mammography**	Reduction in breast cancer mortality Widely accessible Cost-effective for mass screening	Limited sensitivity in dense breast tissue Exposure to radiation False positives
**MRI**	High sensitivity (also in dense breasts) Effective for high-risk populations Improved detection of mammography-occult cancers	High cost Higher rate of false positives No definitive evidence of mortality reduction when used alone for screening
**Ultrasound**	No ionizing radiation exposure Well tolerated Detect small/node-negative cancers Less expensive	High rate of false positives Operator-dependent variability Less consistent than mammography or MRI
**PET**	Functional imaging to detect metabolic activity of tumors and evaluate treatment response Improves accuracy of cancer staging when combined with CT	High cost Complex image interpretation Lower specificity for small lesions and specific tracers Not suitable for routine screening

**Table 2:** Comparative overview of recent studies (2009–2024) on various innovative methodologies for early detection of breast cancer in different biological sample types. Review papers cited in the text have not been included in this table.

Ref.	Technique	Sample	Detection Accuracy	Sensitivity Specificity	Finality	Key Advantages	Key Limitations
[18] Shahrokhian et al., 2018	Electrochemical DNA biosensor	Human serum	LOD: 3 fM	Both high	Detection of BRCA1 gene	Label-free, rapid, economical	Complexity of electrode preparation, interference
[20] Hakimian et al., 2020	Electrochemical biosensor (CV)	Serum	Detection range: 2 10^−20^–2 10^−12^ mol	Ultrasensitive	Detection of miRNA-155	Simple	Biological variability
[21] Hu et al., 2014	Electrochemical bioaptasensor (DPV)	Serum	LOD: 2.2 nM	Both high	Detection of MUC1	High sensitivity, wide linear range	Interference from complex sample components
[22] Zhao et al., 2011	Electrochemical bioaptasensor (ACV)	Human serum and blood	LOD: 5 pM (190 pg/mL)	Both high	Detection of VEGF	Simple preparation, Regenerable, reusable,	Aptamer degradation, matrix interference
[23] Marques et al., 2014	Electrochemical biosensor (LSV)	Human serum	LOD: 4.4 ng/mL	Not specified	Analysis of HER2 ECD	Sensitive, useful for follow-up	Enzyme stability, long analysis time
[24] Zhu et al., 2013	Electrochemical biosensor (SWV)	Human serum	LOD: 0.037 ± 0.002 pg/mL	Both high	Detection of HER2	Ultrasensitive, easy microscopic observation	Surface modification process
[25] Ribeiro et al., 2018	Electrochemical biosensor based on MIP (CV-DPV)	Artificial/spike serum	LOD: <0.10 U/mL	Both high	Detection of CA15-3	Cost-Effectiveness, Stability, Reusability	Interferences, Sample Variability, Quantification Limitations
[26] Majd et al., 2018	FET biosensor (MoS2)	Human serum	LOD: 0.03 fM	Ultrasensitive, High	Detection of miRNA-155	Label-free, good reproducibility	Stability issues with MoS2 and DNA probes
[31] Szymanska et al., 2020	SPRi sensor	Blood plasma	LOD: 0.1 ng/mL	High sensitivity	Detection of CEA	Real-time analysis, high sensitivity	Non-specificity for breast cancer, interference
[32] Erol et al., 2023	NanoMIP–SPR sensor	Human serum	LOD: 11.6 pg/mL	Both high	Detection of HER2	Portable, label-free, rapid, cost-effective	Long-term stability, Specific training and equipment
[33] Verma et al., 2022	PCF-SPR biosensor	Breast cancer cells	MSE: 0.1163	Both high	Detection of MCF-7/MDA-MB-231	High Sensitivity, Machine Learning	Design Parameters, Costs and Complexity
[34] Han et al., 2020	SERS sensor	Human serum (miR-K12-5-5)	LOD: 8.84 × 10^−10^ M	High sensitivity	Detection of miR-K12-5-5p	Enhancement Factor, Uniformity, Stability	Stability, Need for Validation, Cross-reactivity
[35] Wang et al., 2018	FRET-based aptasensor (UCNPs)	Human serum	LOD: 7.9 pg/mL–10.7 pg/mL	Both high	Detection of CEA	Simple configuration, direct operation	Non-specificity for breast cancer, FRET Complexity
[36] Bai et al., 2020	Colorimetric biosensor	DNA extracted from blood	LOD: 10^−18^ M	Both high	BRCA1 mutation	Low cost, rapid, good reproducibility	Weak direct signals at low concentrations
[37] Yang et al., 2017	QCM biosensor	BC cells (tumor tissue)	LOD: 300 cells/mL	High sensitivity	Metastatic potential	High sensitivity, label-free, real-time detection	Low specificity and clinical applicability
[39] Nurlankyzy et al., 2024	Fiber-optic ball resonator sensor	CD44-expressing cancer cells	335 cell/mL	High sensitivity	Early detection of breast cancer	Label-free and fast	Not tested in situ so far
[38] Uliana et al., 2017	μFED	Calf serum	LOD: 10.0 fg/mL	Both high	Detection of ERα	Easy to produce, Multiple Applicability	Limitations in Testing
[40] Gao et al., 2020	Microfluidic chip (three-segment hybridization)	Blood-extracted biomarker	LOD: 1 pM	Both high	Detection of miRNAs	Sensitive, rapid, economical	Requires optimization of hybridization conditions
[41] Du et al., 2023	CUSBr	Breast tissue	LOD: 0.3 cm (cysts); 0.1 cm (anomalies)	Moderate (3 dB contrast)	Monitoring of anomalies/cysts	Non-invasive, standardized, repeatable	Operational skills, Image quality, Long-term stability, skin contact issues
[42] Xu et al., 2016	Piezoelectric Finger array	Model tumors, Gelatin matrix	96% accuracy (46/48 lesions)	High sensitivity	Anomalies in the elastic modulus	Direct measurement of tissue stiffness, in situ	Manual operation, time-consuming, small sample size
[43] Elsheakh et al., 2023	Microwave textile-based antenna sensor	Phantom for breast tissue	89% (Mean CatBoost) accuracy	Both moderate	BC detection/ classification	Non-invasive, wearable	Advanced equipment, Parameter limitations
[44] Elouerghi et al., 2022	Thermography system (bioheat microsensors)	Mammary phantom	Accuracy 0.1 °C	85% sensitivity 90% specificity	Thermal anomalies	Wearable, Non-invasive	Need for robust calibration to ensure accurate readings
[45] Sree et al., 2020	Cyrcadia Breast Monitor	Breast tissue	78% accuracy of ML model	84% sensitivity 72% specificity	Anomaly detection	Machine Learning model, Non-invasive monitoring	Limitations in Data, Need for Further Validation
[46] Yang et al., 2021	E-Nose	Alveolar exhaled breath	91% accuracy	86% sensitivity 97% specificity	BC detection/ classification	Machine Learning, Intra-operative applicability	Influenced by anesthetics, individual variability
[47] Díaz de León-Martínez et al., 2020	E-Nose	Exhaled Breath	98% CDA model accuracy	Both high	Screening for breast cancer	Machine Learning, Non-invasive, early screening	Sensitive to environmental factors, small sample size
[48] Wan et al., 2024	Field-effect transistor (FET)-based biosensor	Saliva	LOD: 1 fg/mL	70/dec HER2, 30/dec CA15-3	Detection of HER2, CA15-3	Non-invasive, rapid, easy to use	Cross-reactivity, need for clinical validation
[49] Wei et al., 2009	Electrochemical biosensor	Saliva	LOD: 3.9 fM (mRNA), 7.4 pg/mL (protein)	90% for both	Detection of IL8 mRNA/protein	Simultaneous detection, Non-invasive	Non-specificity for breast cancer, Limited sample size
[50] Torrente-Rodríguez et al., 2015	Amperometric biosensor	Saliva	LOD: 0.21 nM (mRNA), 72.4 pg/mL (protein)	High sensitivity	Detection of IL8 mRNA/protein	Simultaneous detection, Non-invasive, easy to automate	Non-specificity for breast cancer, cross-reactivity
[51] Benet et al., 2022	E-Nose and GC-MS	Urine	92.31% (GC-MS) 75% (E-Nose)	85.71% (GC-MS) 50% (E-Nose)	Detection of BCs VOCs	Non-invasive, low-cost, Machine Learning	Specificity of sensors, Variability of VOCs
[52] Leemans et al., 2022	GC × GC − MS	Sweat	F1-score: 0.93	1.0 sensitivity 0.8 specificity	Identification of BC- VOCs	Non-invasive, low-cost, Machine Learning	Limited sample size, Variability in VOCs
[53] Elsheakh, D. N. et al.	Werable flexible antenna-based sensors	In situ	Simulation with tumors down to 5–10 mm	NA	Early detection and monitoring	Wearable	Only simulation tests so far
